# Differential immune gene expression associated with contemporary range expansion in two invasive rodents in Senegal

**DOI:** 10.1038/s41598-020-75060-2

**Published:** 2020-10-26

**Authors:** Nathalie Charbonnel, Maxime Galan, Caroline Tatard, Anne Loiseau, Christophe Diagne, Ambroise Dalecky, Hugues Parrinello, Stephanie Rialle, Dany Severac, Carine Brouat

**Affiliations:** 1 CBGP, INRAE, CIRAD, IRD, Montpellier SupAgro, Univ Montpellier, Montpellier, France; 2 Départment de Biologie Animale, Faculté des Sciences et Techniques, Université Cheikh Anta Diop (UCAD), Fann, Dakar, Senegal; 3 LPED, IRD, Aix Marseille Univ, Marseille, France; 4 MGX-Montpellier GenomiX, c/o Institut de Génomique Fonctionnelle, Montpellier, France

**Keywords:** Evolutionary ecology, Invasive species, Molecular ecology

## Abstract

Biological invasions are major anthropogenic changes associated with threats to biodiversity and health. However, what determines the successful establishment and spread of introduced populations remains unclear. Here, we explore several hypotheses linking invasion success and immune phenotype traits, including those based on the evolution of increased competitive ability concept. We compared gene expression profiles between anciently and recently established populations of two major invading species, the house mouse *Mus musculus domesticus* and the black rat *Rattus rattus*, in Senegal (West Africa). Transcriptome analyses identified differential expression between anciently and recently established populations for 364 mouse genes and 83 rat genes. All immune-related genes displaying differential expression along the mouse invasion route were overexpressed at three of the four recently invaded sites studied. Complement activation pathway genes were overrepresented among these genes. By contrast, no particular immunological process was found to be overrepresented among the differentially expressed genes of black rat. Changes in transcriptome profiles were thus observed along invasion routes, but with different specific patterns between the two invasive species. These changes may be driven by increases in infection risks at sites recently invaded by the house mouse, and by stochastic events associated with colonization history for the black rat. These results constitute a first step toward the identification of immune eco-evolutionary processes potentially involved in the invasion success of these two rodent species.

## Introduction

Biological invasions are considered to be one of the five most important anthropogenic changes, with major impacts on biodiversity^1,2^ and human health^3,4^. Considerable progress has been made in determining population histories and the invasive routes of exotic species, but the mechanisms driving the successful establishment and spread of introduced populations remain unclear. In particular, the assertion that invasion success depends on the contemporary evolution of phenotypic traits of advantage at invasion fronts has seldom been tested, particularly in vertebrates (but see^5,6^).

From an eco-evolutionary perspective, invasion success may depend on pre-adaptation within the original range^7,8^ or on rapid changes in phenotypic traits (through selection on new/standing genetic variation or adaptive plasticity) advantageous in newly colonized areas^7,9^. In this context, many hypotheses have been formulated concerning traits related to the ability to cope with new environments^10^. In particular, large changes in parasite pressure can occur in introduced species, because of enemy release or the spillover of native parasites, along invasion gradients between the native and invaded areas^11–15^ or within invaded regions, from introduction areas to invasion fronts^16,17^. Invasion success may therefore depend on immune strategies. Two general hypotheses have been developed, in accordance with the life history theory. The “evolution of increased competitive ability” (EICA)^18^ is based on trade-offs between immune traits and other life history traits. According to this theory, the loss of parasites that often occurs during the course of invasion should lead to a release of energy resources previously dedicated to anti-parasite defenses. The reallocation of this energy to other life history traits, increasing competitive abilities and favoring range expansion (e.g. dispersal, reproduction), would therefore be expected to be favored. According to the “EICA-refined” hypothesis^19^, invaders may encounter new infection risks during the course of invasion, due to a spillover of native parasites, for example. In such situations, the allocation of energy to anti-parasite defenses would be expected to increase to a lesser extent, instead being modulated in favor of defense strategies with lower costs. Indeed, different immune pathways seem to have different developmental, maintenance and use costs^20^. In vertebrates, costly immune pathways, such as inflammation, may be attenuated, whereas less costly pathways, such as antibody-mediated responses, may be favored^5,19,21,22^. As an alternative to both the EICA and EICA-refined hypotheses, it is also possible that invaders display stronger immune responses as a result of exposure to new parasites and their pathologic effects at invasion fronts.

A recent analysis reviewing empirical studies showed that, in most cases, immune responses display variations during invasions, although no general pattern supporting the EICA hypotheses was detected^21^. The authors highlighted a problem due to the lack of studies reporting a large array of immune responses for the correct assessment of immunocompetence and estimation of immune trade-offs. Unfortunately, immune phenotyping is often based on a small number of immune effectors, due to the availability of only small amounts of material (blood, tissue), or the difficulty obtaining immune kits for non-model species.

The recent advent of ‘omics’ technologies has made it possible to explore a large set of genes and pathways involved in the response to new conditions experienced at invasion fronts. Genome scans analyzing genome-wide variation with a view to detect loci evolving under positive directional selection have been successfully performed in some case studies of invasion (e.g.,^23–25^). These scans have identified signatures of contemporary adaptation associated with invasion, some of which are related to immunity^26^. Nevertheless, this genomic approach, based on the detection of single-nucleotide polymorphism outliers, cannot detect rapid adaptation occurring over a few generations, through phenotypic plasticity, for example. Transcriptomics, the analysis of gene expression at a genome-wide scale, is a complementary approach that could potentially fill in these gaps, making it possible to decipher the molecular mechanisms underlying phenotypic changes^27,28^. Indeed, gene expression is an essential mechanism for rapid acclimatization or adaptation to new environments^29–33^. Transcriptomics has thus been used to identify genes and gene regulatory pathways contributing to phenotypic variation at invasion fronts. Evidence for transcriptomic divergence along invasion gradients has already been reported^25,34^, in some cases highlighting the important role of immune regulation in invasion success^35^. However, only one study to date has analyzed immune gene expression in the context of established variations of parasite pressures along invasion routes^36^. Such studies are essential, to test the EICA hypotheses and to increase our understanding of the interactions between immune system regulation and invasion success^21^.

We investigated transcriptional profiles along the invasion routes of the house mouse *Mus musculus domesticus* and the black rat *Rattus rattus,* which are currently invading Senegal (West Africa). Both these rodent species originate from Asia and made use of human migration to expand their distribution range worldwide^37,38^. In Senegal, historical records (see references in^39^ and molecular analyses^40,41^) show that these rodents were first brought to Senegal, by sea, by European explorers and settlers. They were reported to be common in coastal villages and towns from the middle of the nineteenth century, and their distribution areas remained restricted to the coastal area until the beginning of the twentieth century. Both taxa then spread further inland, due to increases in human activities and the further development of transport infrastructures. Our previous study, based on a small number of immune effectors, highlighted the occurrence of stronger inflammatory and/or antibody-mediated responses at recently invaded sites than at anciently invaded ones, for both invaders^42^. These immune variations may reflect responses to new parasite pressures encountered in recently invaded areas. Surveys of the bacterial communities present in the spleens of rodents from Senegal were found to be consistent with this prediction^43^, as changes in bacterial composition were observed along the routes of invasion of both the house mouse and the black rat. Exotic rodents were exposed to a greater diversity of *Mycoplasma* sp. and to higher levels of *Bartonella* sp. at recently invaded sites, due to the high prevalence of these bacteria in the native rodent *Mastomys erythroleucus*^43^. By contrast, decreases in the pressure due to certain parasites were also observed, as the number of helminths present in the gastrointestinal tract of rodents was smaller at recently invaded sites than at anciently invaded ones, suggesting enemy release for these macroparasites^44^. Decreases were observed in both the overall prevalence and species richness of gastrointestinal helminths, for both the house mouse and the black rat^44^.

We therefore developed a whole-RNA sequencing (i.e., RNAseq) approach to assess differential patterns of gene expression between populations from recently invaded and long-established invasion sites. We focused on the spleen in both species, as this immune-related organ of vertebrates is commonly used for immunological studies. We tested the null hypothesis of an absence of difference in immune gene expression patterns along invasion routes (i.e. recently invaded sites *vs*. long-established invasion sites). We tested two alternative hypotheses. According to the EICA-refined hypothesis, expression levels would be expected to be lower for genes encoding proteins involved in energetically costly immune pathways (e.g., inflammation) and higher for genes encoding proteins involved in cost-effective immune pathways (e.g., antibody-mediated responses), at recently invaded sites^19^. The changes in infection risk at recently invaded sites may mediate such trade-offs between energetically costly and cost-effective immune pathways^43,44^. We also tested the alternative hypothesis that rodents from recently invaded sites display higher overall levels of immune gene expression in response to the new parasite pressures encountered^5,45,46^.

More specifically, we investigated the following questions: (1) Does immune gene expression differ between anciently and recently invaded populations of mice and rats? (2) Are certain functional categories of immune genes overrepresented among the differentially expressed genes? (3) Do these results support the EICA hypothesis or the EICA-refined hypotheses? Or are these results consistent with an overall increase in immune gene expression reflecting the new parasite pressures experienced at the invasion front?

## Materials and methods

### Ethics statements

Sampling campaigns were performed on private property with explicit prior agreement from family, or the local authority or institution concerned. None of the rodent species investigated here has protected status (see list of the International Union for Conservation of Nature). All animal-related procedures were performed according to the official ethics guidelines of the American Society of Mammalogists^47^. All protocols presented here were performed with the explicit prior agreement (CBGP: D 34-169-1) of the relevant institutional committee (Regional Head of the Veterinary Service, Hérault, France). They were also performed in accordance with the requirements of Senegalese and French law.

### Sampling sites

**Figure 1:**
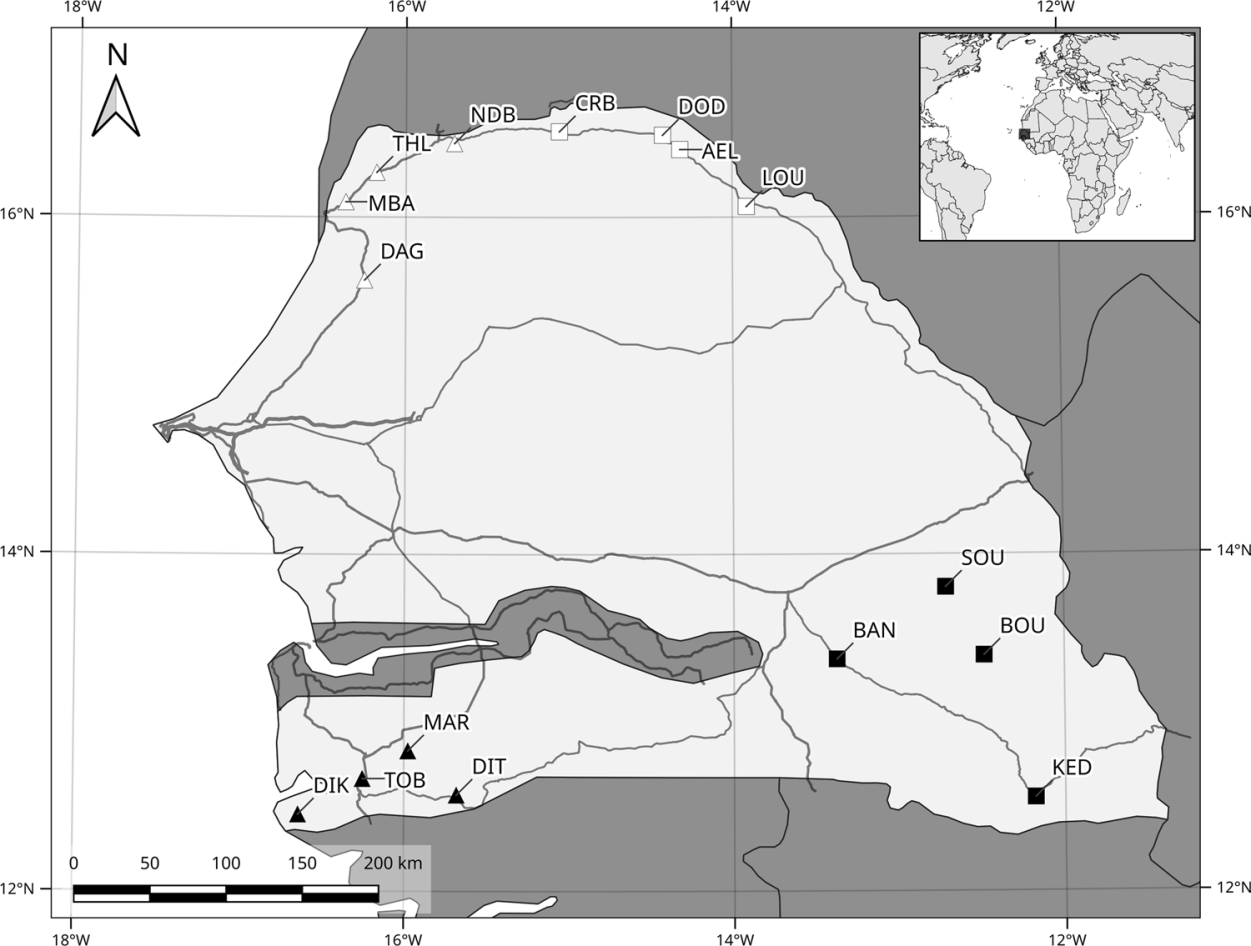
Sampling sites for the house mouse, *Mus musculus domesticus* (symbols in white) and the black rat, *Rattus rattus* (symbols in black) invasion routes. Triangles and squares correspond, respectively, to anciently and recently invaded sites. *DAG* Dagathie, *MBA* Mbakhana, *THL* Thilene, *NDB* Ndombo, *CRB* Croisement Boube, *DOD* Dodel, *AEL* Aere Lao, *LOU* Lougue, *DIK* Diakene Wolof, *DIT* Diattacounda, *MAR* Marsassoum, *TOB* Tobor, *BAN* Badi Nieriko, *BOU* Boutougoufara, *KED* Kedougou, *SOU* Soutouta.

We used the spleens of *M. m. domesticus* and *R. rattus* animals used in a previously described bacterial metabarcoding study^43^. Briefly, live trapping was performed for both these invasive species at four anciently sites and four recently invaded ones (Fig. 1).

No accurate estimate of the time of introduction was available for these rodents, but we used historical records to differentiate between anciently invaded sites and recently invaded sites. Exotic mice and rats were common in the villages and cities of coastal areas of Senegal in the middle of the nineteenth century, but the available records did not provide precise geographic locations (see refs in^39^). We therefore considered coastal areas to have been colonized between 300 and 100 years ago. Regular rodent community surveys have been performed across Senegal since the 1970s. These surveys indicate that, before the 1990s, the house mouse was absent from the villages and cities of the middle of the Senegal River valley, and the black rat was absent from western Senegal (data summarized in^39^). We therefore considered inland sites to have been colonized less than 30 years ago. Previous population genetic studies confirmed that most of the house mouse populations from the north of Senegal belonged to the same genetic group, which colonized northern Senegal from Saint Louis^41^. Black rat populations from south-eastern Senegal form a separate genetic group, which originated from the coastal area south of Dakar^40^.

### Sample collection and RNA extraction

Fieldwork was conducted during the dry season*,* which runs roughly from October to June in northern Senegal and from November to May in southern Senegal. Mice and rats breed year-round in Senegal, as generally reported in commensal conditions (see refs in^48^), but we chose to define a standardized sampling period, to avoid the possibility of confounding factors associated with seasonal variations. Rodents were killed by cervical dislocation at approximately the same period of the day for all individuals (11 a.m.–2 p.m.). Autopsies were performed and the spleens were collected and immediately placed in RNAlater, stored at 4 °C overnight and then at − 20 °C until further analyses. Only adult rodents (mass > 9 g and/or length > 72 mm for house mice, mass > 54 g and/or length > 145 mm for black rats, see^48^) were included in the analyses, as individual age (at least juveniles vs adults) is known to have a strong influence on immune responses^49^. None of the trapped rodents displayed any sign of disease.

We considered 20 adult rodents per site for all but five sites at which only 14–18 individuals were available (see Table 1). Total RNA was extracted from approximately 5 mg of spleen from each sample with the AllPrep 96 DNA/RNA Kit (Qiagen). The quality and quantity of the purified RNA was assessed by gel electrophoresis and on a NanoDrop spectrophotometer (Thermo Scientific) before pooling, and with a Bioanalyzer 2100 (Agilent) or Fragment Analyzer (Advanced Analytical) after pooling (see below). The RNA integrity (RIN) or quality (RQN) numbers of each pool are provided in Fig. S1. For each rodent species and each site, we constituted two pools of individuals. These two pools contained similar numbers of individuals (from 6 to 10) and had a balanced sex ratio (Table 1). For a given site, the two pools were considered to be ‘biological replicates’ of the site concerned. We therefore constituted 16 equivalent pools of individuals (Table 1). This design made it possible to attain the number of replicates recommended in gene expression analyses for which high levels of variability between replicates are expected (between 6 and 12 replicates per category, see^50^).

**Table 1:** Sampling sites and their invasion-related categorization as well as rodent sample sizes included in each biological replicate along the invasion routes of the house mouse and of the black rat.

Invasion category	House mouse		Black rat	
	Sites	N (M/F)	Sites	N (M/F)
Anciently invaded sites (> 100 years)	Dagathie		Diakene Wolof	
	Pool of RNA #1	10 (5/5)	Pool of RNA #1	6 (3/3)
	Pool of RNA #2	8 (4/4)	Pool of RNA #2	8 (4/4)
	Mbakhana		Diattacounda	
	Pool of RNA #1	10 (5/5)	Pool of RNA #1	10 (5/5)
	Pool of RNA #2	10 (5/5)	Pool of RNA #2	10 (5/5)
	Thilene		Marsassoum	
	Pool of RNA #1	8 (4/4)	Pool of RNA #1	10 (5/5)
	Pool of RNA #2	8 (4/4)	Pool of RNA #2	10 (5/5)
	Ndombo		Tobor	
	Pool of RNA #1	10 (5/5)	Pool of RNA #1	6 (3/3)
	Pool of RNA #2	10 (5/5)	Pool of RNA #2	8 (4/4)
Recently invaded sites (< 30 years)	Croisement Boube		BadiNieriko	
	Pool of RNA #1	10 (5/5)	Pool of RNA #1	8 (4/4)
	Pool of RNA #2	10 (5/5)	Pool of RNA #2	8 (4/4)
	Dodel		Boutougoufara	
	Pool of RNA #1	10 (5/5)	Pool of RNA #1	8 (4/4)
	Pool of RNA #2	8 (4/4)	Pool of RNA #2	8 (4/4)
	Aere Lao		Kedougou	
	Pool of RNA #1	10 (5/5)	Pool of RNA #1	8 (4/4)
	Pool of RNA #2	10 (5/5)	Pool of RNA #2	8 (4/4)
	Lougue		Soutouta	
	Pool of RNA #1	10 (5/5)	Pool of RNA #1	10 (5/5)
	Pool of RNA #2	8 (4/4)	Pool of RNA #2	10 (5/5)

*N* total number of rodents included, *M* number of males, *F* number of females.

### cDNA library preparation and RNA sequencing

cDNA library construction, sequencing and the alignment of filtered read sequences were performed at the MGX platform. RNA-Seq libraries were constructed with the Truseq stranded mRNA sample preparation (low-throughput protocol) kit from Illumina. We used 1 µg of total RNA for library construction. We first purified poly-A-containing mRNA molecules with a poly-T oligomer attached to magnetic beads (poly-A-based mRNA enrichment). The mRNA was then fragmented into small pieces with divalent cations at high temperature. The cleaved RNA fragments were used to generate first-strand cDNA with actinomycin D, random hexamer primers and SuperScript II reverse transcriptase for *M. m. domesticus* or SuperScript IV reverse transcriptase for *R. rattus*. The second strand of the cDNA was synthesized by replacing dTTP with dUTP. These cDNA fragments had an additional single ‘A’ base and were subsequently ligated to the adapter. The products were then purified and enriched over 15 cycles of PCR. The final cDNA libraries were validated and quantified with a KAPA qPCR kit.

For *M. m. domesticus*, four cDNA libraries per sequencing line were pooled, in equal proportions, denatured with NaOH and diluted to 7 pM before clustering. Cluster formation was performed on a Flowcell V3, primer hybridization and 50 cycles of single-end read sequencing were performed on a cBot and a HiSeq2000 (Illumina, San Diego, CA), respectively. For *R. rattus*, four cDNA libraries per sequencing line were pooled, in equal proportions, denatured with NaOH and diluted to 8 pM before clustering. Cluster formation was performed on a Flowcell V4, primer hybridization and 100 cycles of single-end read sequencing were performed on a cBot and a HiSeq2500 (Illumina, San Diego, CA) ,respectively. We performed 100 cycles of single-end sequencing for *R. rattus,* as the only published genome sequence available for rats is from another species, *Rattus norvegicus*.

### Transcriptome analysis

#### Sequence alignment and gene quantification

Image analyses and base calling were performed with Illumina HiSeq Control Software and the Real-Time Analysis component. Demultiplexing was performed and Fastq files were generated with Illumina conversion software (Casava 1.8.2 for the house mouse data, bcl2fastq 2.17 for the black rat data). The quality of the raw data was assessed with FastQC from the Babraham Institute and the Illumina software SAV (Sequencing Analysis Viewer).

A splice junction mapper, TopHat^51^ (v2.0.9 for the mouse data, v2.0.13 for the black rat data), using Bowtie^52^ (v2.1.0 for the mouse data, v2.2.3 for the black rat data), was used to align the reads to the *M. musculus* genome (UCSC mm10) or the *Rattus norvegicus* genome (UCSC rn4) with a set of model gene annotations (genes.gtf downloaded from UCSC on March 6, 2013 for the mouse genome, on July 17, 2015 for the brown rat genome). Final read alignments containing more than three mismatches (house mouse) or nine mismatches (black rat) were discarded. Again we used a different threshold for mouse and rat data, as the reference genome sequence for rat was that of *Rattus norvegicus*, and genomic studies have shown that these two species diverged about 2.9 million years ago^53^. Read alignment rates were above 83.3% for all *M. m. domesticus* libraries and above 74.0% for all *R. rattus* libraries. Samtools (v0.1.18 for the mouse data, v1.2 for the black rat data) was used to sort the alignment files. Genes were then counted with HTSeq count (v0.5.4p5 for the mouse data, v0.6.1p1 for the black rat data), in union mode^54^. The data were obtained in a strand-specific assay, so the read had to be mapped to the opposite strand.

#### Analysis of differential gene expression between populations

Differentially expressed (DE) genes were identified with the Bioconductor^55^ package edgeR v3.4.0, under R 3.0.2 for the mouse data, v3.8.6 under R 3.2.3 for the black rat data^56^, and limma^57^. Data were normalized with relative log expression (RLE)-normalization factors^58^. Genes with fewer than 10 occurrences (reads) were filtered out and removed before the statistical analysis. This made it possible to limit the number of statistical tests and, therefore, to reduce the impact of correction for multiple testing.

Two statistical factors were included in the experimental design: the main factor included was the timing of the invasion (two levels: recent and ancient invasion), and the second factor was the site at which the rodent was caught (eight sites in total: four recently invaded sites and four anciently invaded ones). For each site, we considered two biological replicates (i.e. the two pools of individuals with a balanced sex ratio and containing a similar number of individuals).

We used three analytical strategies to identify the differentially expressed genes. For all strategies, the *p* value threshold was set to 0.05 after Benjamini–Hochberg correction for multiple testing. We first retained only one randomly chosen biological replicate per site and compared the four recently invaded sites with the four anciently invaded ones (the ‘4vs4 approach’, see Suppl. Fig. S2). This strategy made it possible to remove the second factor (site) from the analysis. As there was no particular reason to retain either the first or the second replicate for a given site, we analyzed all 256 possible combinations of replicate selection. We then considered all sites and replicates for each invasion period (recent vs. ancient invasion) together (the ‘8vs8 approach’, see Suppl. Fig. S2). The factor “site” was included in a generalized linear model (GLM) to take into account the use of two biological replicates for each site.

Considerable variability was observed between the replicates of a given site (Suppl. Fig. S3a, b), rendering the ‘4vs4 approach’ highly conservative, but potentially resulting in false positives when the ‘8vs8 approach’ was applied. Combining the results of the ‘4vs4 approach’ and the ‘8vs8 approach’ therefore seemed to be the most prudent choice, providing a maximum robustness. The genes identified with the ‘8vs8 approach’ and found to be differentially expressed in more than 85% of the 256 combinations of the ‘4vs4 approach’ were considered to be DE. The threshold of 85% was fixed based on a bar plot of the number of ‘4vs4’ comparisons in which a gene (highlighted in the ‘8vs8 approach’) was found to be differentially expressed.

Finally, we analyzed gene expression with limma and the *duplicateCorrelation* function^57^. We fitted a generalized linear model to the data, including site as a random effect within invasion categories. This approach corresponds to a random effects model, except that genes are constrained to share the same within-category correlation^57^.

### Functional and enrichment analyses

*M. m. domesticus* and *R. rattus* genes were functionally annotated with gene ontology (GO) terms with Search Tool for the Retrieval of Interacting Genes/Proteins (String) v10.5^59,60^. We assessed gene enrichment by performing Fisher’s exact tests on the sets of DE genes from house mouse and black rat, house mouse *M. musculus* and brown rat *R. norvegicus* GO as the background and String software. We corrected p values for multiple testing by the Benjamini and Hochberg false discovery rate (FDR) method. GO terms with a FDR *p* value < 0.05 were considered to be significant. The redundancies of significantly enriched GO terms were reduced with REVIGO^61^, using a similarity cutoff of 0.7. We also performed enrichment analyses on biological processes and biological pathways supplied by KEGG (https://www.genome.jp/kegg/pathway.html), implemented in String.

Finally we performed a global protein network analysis based on DE genes, using a String database including interaction databases (including both direct (physical) and indirect (functional) associations), genetic interactions and shared pathway interactions. This analysis revealed the interactions between key components of different pathways. Two PPI networks were constructed by mapping all DE genes and immune-related DE genes, respectively, to the STRING database, with confidence scores > 0.7 (high level). PPI networks were visualized and analyzed in Cytoscape software, via the web interface of String. Enrichment in these protein–protein interactions was assessed by performing Fisher’s exact tests with correction for multiple testing (FDR).

## Results

### Qualitative description of expression patterns

Thirty-two transcriptome libraries were sequenced (see Table 1 for details). These libraries produced a mean of 51.6 million paired reads. The numbers of reads retained after filtering ranged from 37.2 to 76.7 M, corresponding to 92.82% of all reads. For the house mouse (3 mismatches/paired read allowed), 85.68% of the reads passing the quality filter mapped to the *M. musculus domesticus* reference genome, and 1.58% of these reads mapped to multiple regions. For the black rat (*R. rattus,* for which we allowed 9 mismatches/paired read with the *R. norvegicus* reference genome), 81.66% of the reads passing the quality filter were mapped to the reference genome of the related *R. norvegicus*, and 0.98% of these reads mapped to multiple regions of the reference genome.

### Differential expression (DE) analyses

Along the mouse invasion route, 18 of the 17,853 filtered genes tested with edgeR and the ‘4vs4 approach’ were systematically found to be differentially expressed between recently and anciently invaded sites. For the 16,630 filtered genes tested with the ‘8vs8 approach’, we detected 593 differentially expressed genes, 364 of which were found in at least 85% of the 256 ‘4vs4 approach’ comparisons. Five genes were detected by both the ‘4vs4’ and ‘8vs8’ approaches (*Hal, Rnf183, Serpina6, Wif1, 9030619P08Rik*; see details in Suppl. Table S1a).

Along the black rat invasion route, 54 of the 13,190 filtered genes were systematically differentially expressed between recently and anciently invaded sites with the ‘4vs4 approach’. We found 268 DE genes among the 12,747 filtered genes analyzed with the ‘8vs8 approach’, 83 of which were detected in at least 85% of the ‘4vs4’ comparisons. Forty-two differentially expressed genes were detected by both the ‘4vs4’ and ‘8vs8’ approaches. Detailed results are shown in Suppl. Table S1b.

**Figure 2:**
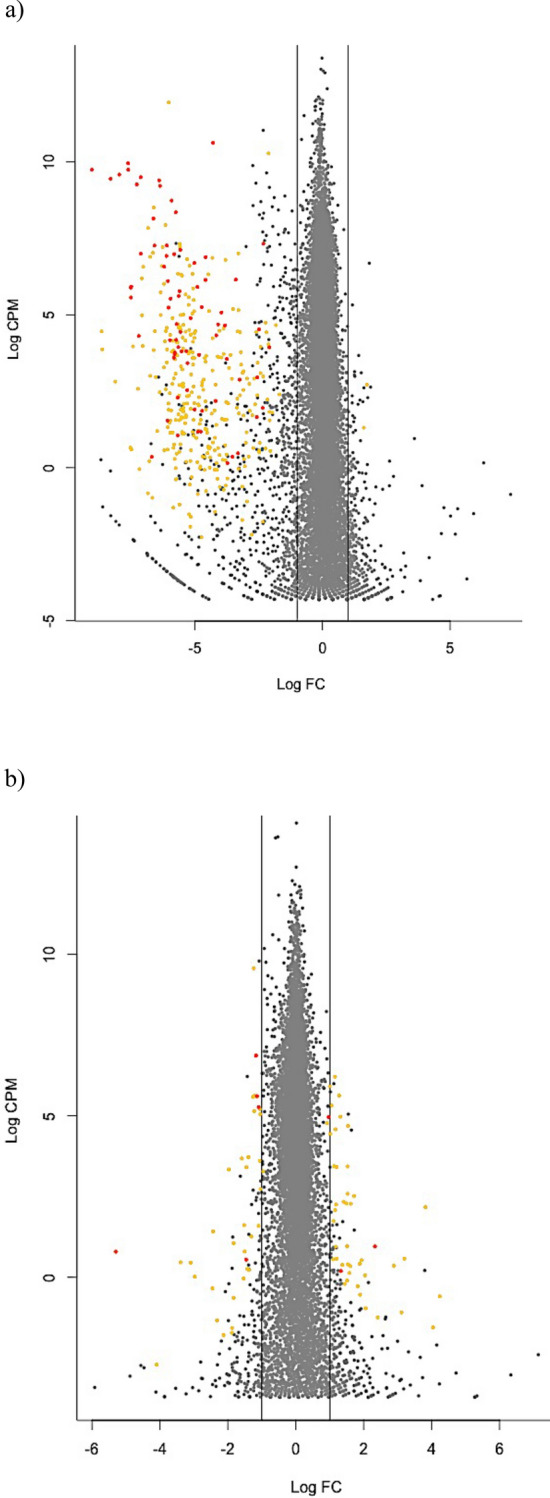
Significantly differentially expressed genes (orange dots) and immune related genes (red dots) between anciently and recently invaded sites along the invasion routes of (**a**) the house mouse and (**b**) the black rat. Only the 73 immune related genes belonging to biological pathways found to be significantly enriched are represented for the house mouse. Vertical lines indicate 1 log fold change (Log FC). The x-axis indicates genes that are down- (negative values) and up-regulated (positive values) in anciently compared to recently invaded areas.

We focused additional analyses on these 364 and 83 genes identified as DE in the ‘8vs8’ approach and detected in at least 85% of the ‘4vs4’ comparisons, for the house mouse and black rat, respectively. The magnitude of expression differences differed significantly between mouse and rat, with higher ‘log fold changes’ observed for mouse genes (Fig. 2, Wilcoxon test, *p* = 0.01). The log fold change (LogFC) per gene ranged from − 9.04 to 2.34 for the mouse and from − 5.79 to 4.49 for the rat DE genes. Mean coverage ranged from − 3.16 to 11.94 LogCPM (counts per million) for the mouse and − 2.41 to 9.56 LogCPM for the rat DE genes.

When site was included as a random effect in the generalized linear model fitted with limma, we found no gene differentially expressed between the anciently and recently invaded sites along the house mouse invasion route. Along the rat invasion route, 12 genes were differentially expressed between recently and anciently invaded sites. All of these sites were also identified with edgeR (‘8vs8 approach’), and 11 were also found in at least 85% of the 256 ‘4vs4 approach’ comparisons.

### Functional analyses of DE genes

For the house mouse dataset, String provided at least one gene ontology (GO) annotation for 345 of the 364 DE genes (Suppl. Table S1a). Of the 2098 GO annotations identified, 357 GO terms corresponding to biological processes displayed significant enrichment (FDR < 0.05) relative to the house mouse reference genome. In particular, 10 immune-related functions were significantly enriched, including inflammatory (GO:0006954), humoral (GO:0002455, GO:0006959), and innate (GO:0045087, GO:0034097) responses or activation/regulation of immunity (GO:0006952, GO:0002253, GO:0050776). We also detected enrichment for two biological processes relating to the response to wounding (GO:0009611, GO:1903035). These enriched GO terms relating to immunity corresponded to 73 DE genes, 20% of all the DE genes (see Suppl. Table S1a). The REVIGO analysis yielded 20 main clusters for the category ‘Biological Process’, taking redundancies into account. Inflammation and acute-phase response were part of the second most represented cluster (Suppl. Fig. S4). Using the Kegg algorithm, we identified 47 pathways as enriched. We found that 29 immune-related genes belonged to these over-represented Kegg biological pathways, the most significant of which was immune ‘Complement and coagulation cascades’ (FDR = 1.56e−30). Note that considering differentially expressed genes as the background for these analyses did not affect these results, as we also observed several significantly enriched GO terms corresponding to immune responses, acute-phase and humoral responses being the second main clusters for the category ‘Biological Process’ while taking into account redundancies with REVIGO and ‘Complement and coagulation cascades’ being the most significantly over represented biological pathways using Kegg algorithm.

**Figure 3:**
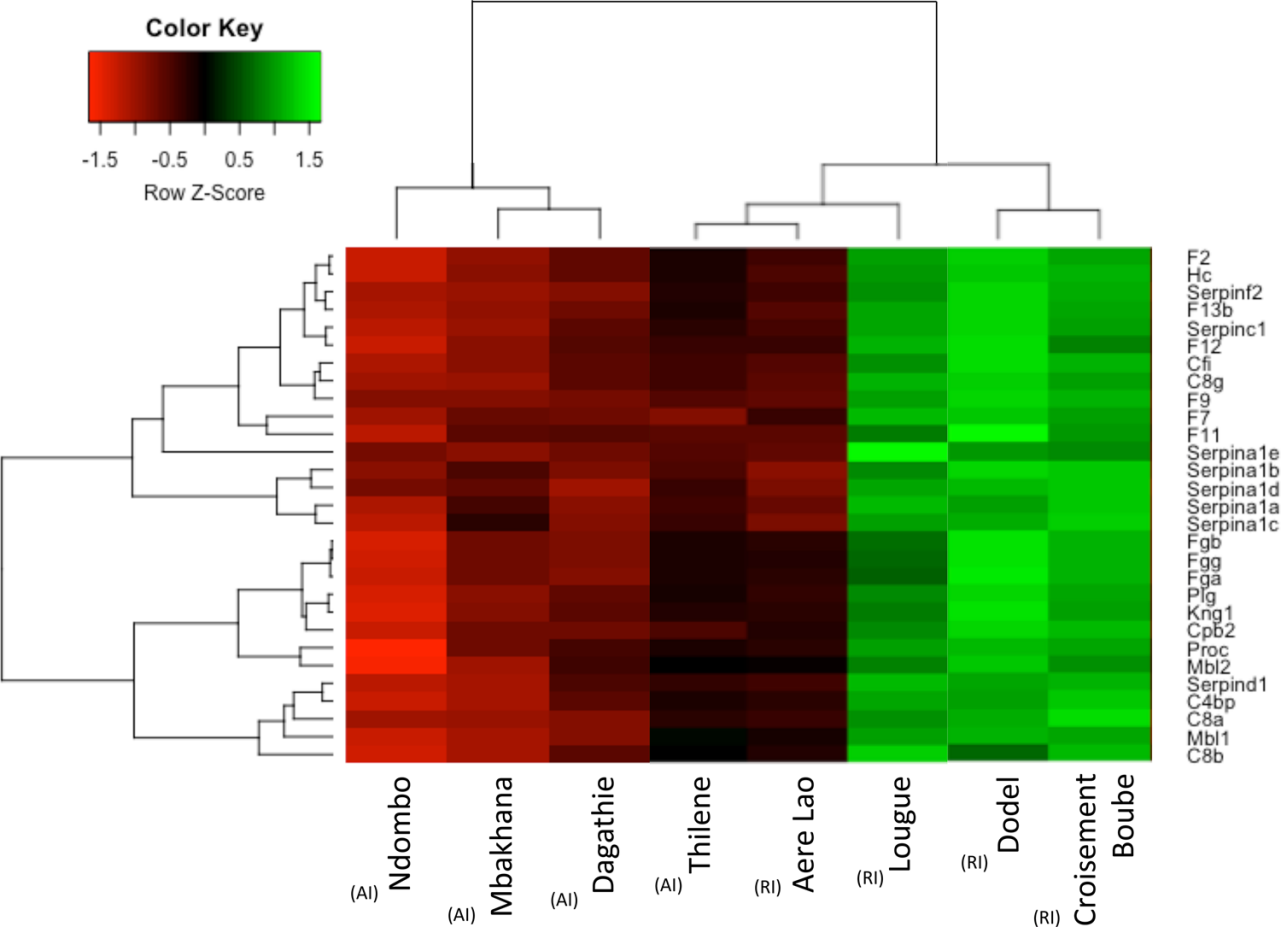
Heatmap of the differentially expressed (DE) genes between the anciently and recently invaded sites of the house mouse (*M. musculus domesticus*). The normalized read counts for the expressed genes are shown. For clarity, the heatmap was built in R using heatmap.2 for 29 immune related genes belonging to over-represented KEGG biological pathways. The genes (rows) and samples (columns) were clustered using dendrograms built with Ward distance and hierarchical clustering. Anciently and recently invaded sites are indicated using (AI) and (RI), respectively.

Interestingly, the distribution of log fold-change (LogFC) for these immune-related genes corresponding to enriched GO terms or pathways was shifted towards higher values than for the 364 DE genes (Wilcoxon test, *p* = 1.52 × 10^–3^; Suppl. Fig. S5). The heatmaps built on the normalized read counts for the immune-related genes belonging to the biological processes (and pathways) found to be overrepresented revealed a downregulation of these genes at all anciently invaded sites, and an upregulation for all recently invaded sites except Aere Lao (Fig. 3, Suppl. Fig. S6). In this last population, the expression pattern observed was similar to that at anciently invaded sites.

**Figure 4:**
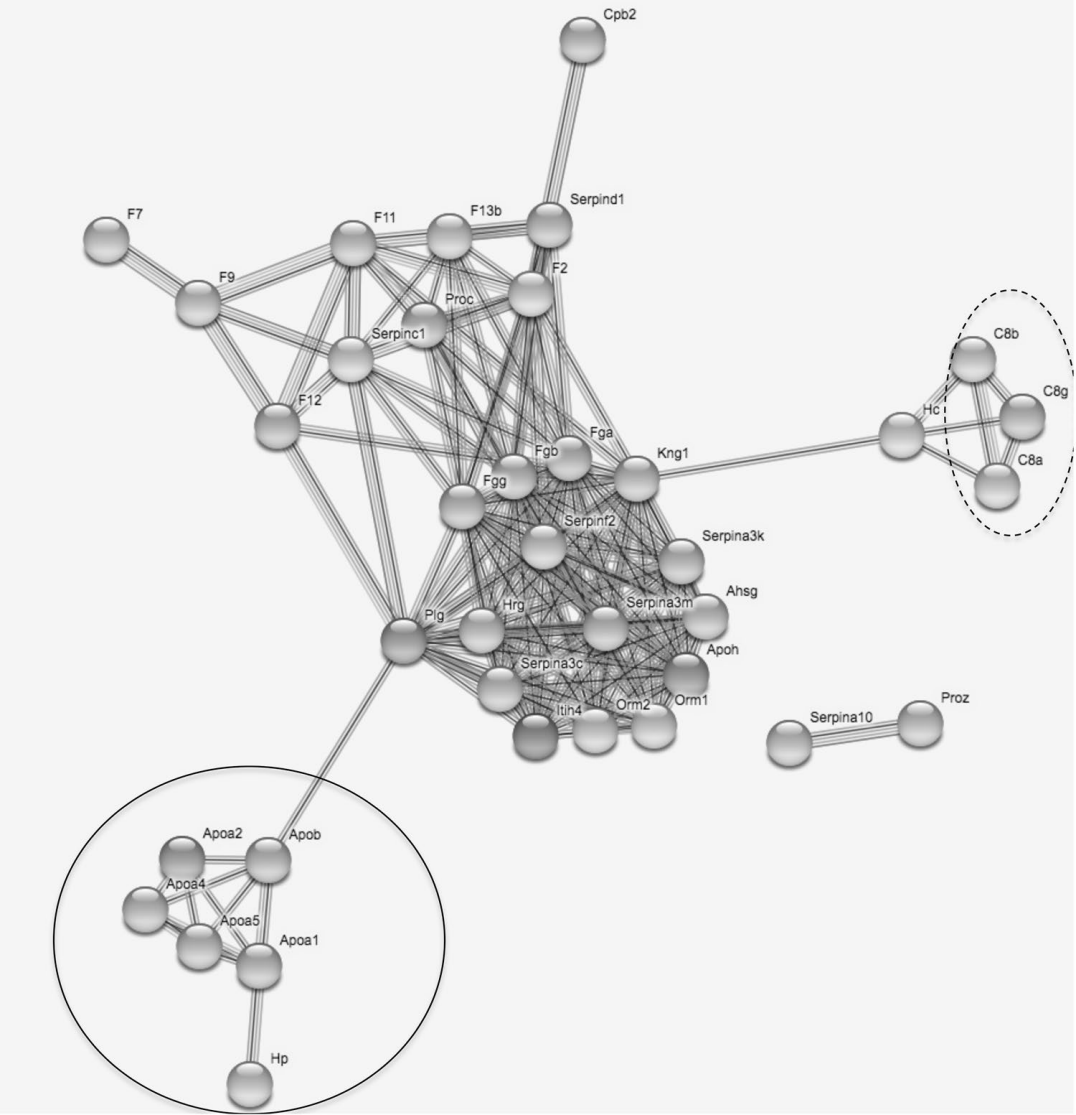
Protein–protein interaction network drawn from the 73 immune genes found to belong to enriched biological processes related to immunity. Nodes correspond to proteins, the thickness of edge network indicates the strength of data support, and the minimum required interaction score was set to 0.7 (high confidence level). The plain circle indicates the alipoprotein A–aloa—and haptoglobin–Hp–protein. The dashed circle indicates the complement proteins (C8). Fibrinogen (Fg) and serine peptidase inhibitor (serpin) proteins are distributed within the highly connected central part of the network.

Finally, using String, we showed that the protein–protein interaction (PPI) network based on the 364 DE genes was significantly enriched (*p* < 1.0e−16, 731 edges found, 54 expected), indicating a larger number of interactions between these proteins than would be expected for a set of proteins of similar size drawn at random from the sequences of the mouse genome. Two main clusters were detected, one including cytochrome P_450_ and UDP glucuronosyltransferase proteins, and the other consisting mostly of immune-related proteins, including fibrinogen, serin peptidase inhibitor (serpin), apolipoprotein and complement proteins. The PPI interaction network built upon the restricted dataset including the 73 immune-related genes belonging to the biological processes shown to be overrepresented also displayed significant enrichment (*p* < 1.0e−16, 561 edges found, 13 expected). The network had a hierarchical structure, with the main cluster including the fibrinogen and serine peptidase inhibitor (serpin) proteins, and two other clusters of highly connected proteins, one including apolipoprotein A and haptoglobin, and the other, complement proteins (Fig. 4).

For the *R. rattus* dataset, GO annotations were available for 75 of the 83 DE genes identified (Suppl. Table S1b). Eight of these annotated DE genes related to immune biological processes (i.e., 10.66% of all DE genes; Suppl. Table S1b). We identified no significantly enriched biological process or pathway, even when redundancies were considered with Revigo. The protein–protein interaction network built from String displayed no significant enrichment (*p* = 0.09). Similar conclusions were obtained when the dataset was restricted to the 12 genes found to be differentially expressed, with site considered as a random effect within invasion category.

## Discussion

We present here a comparative transcriptomics approach based on the sequencing of spleen RNA, for two invasive rodent species studied along their invasion routes in Senegal. We aimed to detect adaptive divergence between recently and long-established rodent populations that could at least partly account for invasion success. More specifically, we investigated the differential expression of immune-related genes, to determine whether the patterns of expression observed supported either the EICA or the EICA-refined hypotheses.

Invasion success is thought to be dependent on a shift in phenotypic traits of advantage at invasion fronts, where species are likely to be exposed to new environmental conditions^9,10,62^. Using comparative transcriptomics in wild populations, we show here that a phenotypic shift^63^ has occurred along the mouse and rat invasion routes in Senegal. Indeed, we detected variations in gene expression levels between recently and long-established rodent populations. Climatic variations and changes in habitat types covary with invasion categories along the invasion routes of both mouse and rat^44^, and all the processes that may explain the phenotypic shift may be influenced by environmental factors. This phenotypic shift may reflect stochastic changes due to population history, including founder events and genetic drift^9^, or non-adaptive responses to environmental cues. Alternatively, this phenotypic shift may result from adaptive processes, including phenotypic plasticity^64^ or natural selection^65^. Invasive populations have a lower genetic diversity than their source populations, due to founder events, but may still evolve rapidly through natural selection from the remaining standing genetic variation or de novo mutations^66^. Several experimental immune challenges have been performed, in controlled conditions, on cane toads from parents collected from long-established populations and invasion front populations from Australia. These experiments showed a rapid evolution of immunity in response to the selection pressures occurring during biological invasions^67,68^. Variations of gene expression at invasion front would also be mediated by phenotypic plasticity^69,70^.

Statistical analyses of transcriptomic data for natural populations are not trivial. We therefore used a combination of three analytical strategies to identify differentially expressed genes. It was tempting to consider site as a random effect within invasion category, to account for the inherently nested structure of our experimental design. However, the strong variability of gene expression between sites made it difficult to detect differentially expressed genes with this strategy. Several lines of evidence suggested that sites within invasion categories could not really be considered as true replicates and that timing of invasion might be variable, according to the history of connections between the site concerned and colonial cities or areas previously invaded by exotic rodents. For instance, colonial settlements were established at some anciently invaded sites (NDB, MBA), but not at others (DAG, THL). These sites without colonial settlements may have been invaded a little later by the house mouse. Historical data also suggest that the Aere Lao site may have been colonized by the house mouse earlier than other sites of the recently invaded category. Indeed, Aere Lao was not connected to the road network until the 1970–1980s, when a weekly rural market was established at this site, creating commercial links with cities already invaded by the house mouse (e.g. Saint Louis, Dakar). This may have favored the introduction of the house mouse at this site 10 or 20 years before this rodent reached the neighboring villages^71^, but several dozens of years after long-standing invasion sites. This history may explain why immune phenotypes in Aere Lao were more similar to those observed in long-established populations from western Senegal for both analysis strategies, and in terms of immune effectors (complement; haptoglobin), as previously shown by immune challenges^42^.

In the house mouse, 20.0% of the genes found to be differentially expressed with a combination of the 4*vs*4 and 8*vs*8 strategies were annotated with functions relating to immunity. We also found that some functional categories of genes were overrepresented among these differentially expressed genes. In particular, we obtained strong evidence for a significantly biased variation of immune gene expression towards an upregulation of serpins, alipoproteins, complement and pro-inflammatory cascades at recently invaded sites. These results were consistent with our previous functional studies^42^, in which immune challenges revealed that antibody-mediated (natural antibodies and complement) and inflammatory (haptoglobin) responses were stronger in mice sampled from recently invaded sites than in those sampled in anciently invaded ones. Our findings therefore support the hypothesis that parasite-mediated selection could contribute to phenotypic differentiation along invasion routes, potentially in interaction with other environmental factors. However, they are not consistent with the EICA hypothesis or the EICA-refined hypothesis^19^. Despite the enemy release patterns detected for mouse helminths in Senegal from sites of long-term invasion to recently invaded sites (decrease in specific richness and overall prevalence, see^44^), we found no evidence of a global decrease in immune-related gene expression at recently invaded sites (the pattern expected according to the EICA hypothesis). We also observed no change in immune-related gene expression that could be attributed to the relative energy costs of immune pathways (the pattern expected according to the EICA-refined hypothesis). Indeed, both inflammation (considered costly in terms of energy) and complement (considered cost-effective in terms of energy) pathways were upregulated^20^. The upregulation of all the immune genes found to be differentially expressed at sites recently invaded by the house mouse therefore strongly supported the alternative hypothesis of an increase in overall infection risk at recently invaded sites. More specifically, the biological processes and pathways displaying significant enrichment, and the protein–protein interaction network, highlighted innate immune responses, which are essential for front-line defense against infections, including bacterial infections. Complement is a protein complex activated immediately after the detection of infection that can induce bacterial lysis^72^. Serine protease inhibitors (serpin) are involved in modulating many physiological functions, including infection and inflammation^73,74^. Apolipoprotein A is involved in lipid transport, and in diverse antimicrobial activities, including the inhibition of bacterial growth and the suppression of virual replication^75^. As such, an increase in expression of the genes encoding these proteins should make it possible for the invading organism to fight effectively against the new bacteria encountered at recently invaded sites^43^. Interestingly, some of these proteins may also have anti-inflammatory activity, through the secretion of particular cytokines (e.g. haptoglobin, serpinG1, C1q, C3a^72,73,76,77^). This function may be particularly important at recently invaded sites, for decreasing the immunopathological effects of inflammation in response to exposure to new parasites^78^. Moreover, the overexpression at invasion fronts of genes encoding anti-inflammatory proteins may counteract the helminth release observed at recently invaded sites along the invasion route of the house mouse^44^. Indeed, as several helminth infections are known to activate regulatory responses that reduce inflammation^79^, helminth release could exacerbate the risk of diseases associated with inflammatory responses.

Similar rapid changes in immunity have been observed in other invading organisms, but these studies revealed a wide array of patterns from which it was not possible to draw any firm conclusions about the potential generic/specific nature of EICA hypotheses^21^. Further studies are required, on a wide range of immune effectors, based on ‘omics methods’^80^, to improve our understanding of the relationships between biological invasion and immunity.

Concerning *R. rattus*, the observed changes in gene expression levels were smaller than those observed in the mouse, and no particular pathway (including immune-related pathways) was significantly overrepresented. The absence of a clear pattern suggested that stochastic events might have strongly shaped the phenotypic variations observed along the route of *R. rattus* invasion. However, caution is required in interpreting our results, which may be due to the limitations of bioinformatics methods (transcriptome annotation was less efficient for *R. rattus* than for *M. musculus domesticus,* as we used the *R. norvegicus* transcriptome as a reference). The eight immune-related genes found to be differentially expressed encode proteins that interact with bacteria (*Rnase6*, *Ptprz1*), viruses (*Ltc4s*, *Hspa1b*) or parasites (*Gfi1b*). Some were downregulated at recently invaded sites (e.g., *Rnase6*)*,* whereas others were strongly upregulated (e.g., *Ptprz1*). No specific pattern has yet emerged concerning potential variations in infection risks. Variations of immune phenotypes, as reflected in gene expression data for the spleen, were, therefore, unlikely to be the main driver of invasion success in the black rat (assuming that invasion success depends on a rapid shift in phenotypic traits in newly colonized areas rather than pre-adaptation in the range of long-standing invasion). Interestingly, we also found, in a previous functional immunity study, that differences in immune responses between anciently and recently invaded sites were less marked for the rat than for the mouse^42^. It was also very difficult to identify any other biological processes that might reflect life history trait adaptation with regard to invasion. The processes found to be associated with differentially expressed genes covered a wide range of functions, including the clustering of sodium voltage channels, the downregulation of cell–matrix adhesion and protein dephosphorylation and the response to cyclic adenosine monophosphate (cAMP). This last function is of potential interest with respect to rat invasion success, because cAMP plays a key role in regulating insulin and glucagon secretion^81^. Variations in cAMP gene expression may, therefore, mediate differences in response to stressful situations, including starvation or fight-or-flight response^82^. It would be interesting to analyze whether such differences could result in different behavioral phenotypes between rats trapped at anciently invaded sites (expected to have a poor performance and high stress response in new environments) and those caught at sites of recent invasion (expected to have a high response capacity in new environments)^83^. It therefore appears particularly important to perform additional transcriptomics analyses on other organs and tissues, to identify the phenotypic changes and ecoevolutionary processes linked to the invasion success of the black rat. The brain, in which key genes underlying behavioral invasion syndrome would be expected to be expressed, may be a relevant organ for future studies^84^.

In conclusion, our work revealed changes in transcriptomic profiles along invasion routes for both the house mouse and the black rat. It is likely that different processes, including colonization history and/or alternative mechanisms by which species adapt to new environments, mediated the invasion success of these two rodent species. The patterns observed could potentially be driven by an increase in infection risks at recently invaded sites for the house mouse and stochastic changes for the black rat. It would be interesting to take into account the potential variability of these patterns with regard to the tissue or organ targeted. Different results might have been obtained if we had targeted other lymphoid organs, lymphatic tissues or non-immune targets. Additional genomic studies and experimental work^25,28,67,68,85^ are required to determine whether the observed differences in gene expression were driven by phenotypic plasticity or directional selection during or after invasion, or whether they reflect the colonization history of the rodents concerned. Moreover, it is also important to determine whether these changes in phenotypic traits influence ecological dynamics (e.g.^86^) and, in turn, invasion success.

## Supplementary information

Supplementary Figures.

Supplementary TableS1.

Supplementary TableS2.

## Data Availability

All sequence data, including raw read sequences and assemblies have been deposited on Geo. Gene ontologies for DE genes are provided in Supplementary Tables 1 and 2. Differential expression data are available from the corresponding author on request.
