# Supplementary material for: Differential immune gene expression associated with contemporary range expansion in two invasive rodents in Senegal
Source: Sci Rep. 2020 Oct 26;10:18257. doi: 10.1038/s41598-020-75060-2 (PMC7589499; doi:10.1038/s41598-020-75060-2)
Supplement: Supplementary file 1 — Supplementary Figures. [file 41598_2020_75060_MOESM1_ESM.pdf]

Differential immune gene expression associated with contemporary range expansion in two invasive rodents in Senegal

Nathalie Charbonnel[1*], Maxime Galan[1], Caroline Tatard[1], Anne Loiseau[1], Christophe Diagne[2,3], Ambroise Dalecky[4], Hugues Parrinello[5], Stephanie Rialle[5], Dany Severac[5] and Carine Brouat[2]

SREP-19-19999A-revised.

Supplementary Figures

Supplementary Information

Figure S1. Bioanalyzer (house mouse) and Fragment analyzer (black rat) profiles detailing the quality and quantity of RNA for each pool a) for *Mus musculus domesticus* samples and b) for *Rattus rattus* samples.

Figure S2. Schematic representation of the '4x4' and '8x8" statistical approaches to detect the genetically differentiated genes.

Figure S3. Pairwise comparisons of the RLE-normalized total number of reads between libraries for a) the house mouse and b) the black rat. For the house mouse, anciently invaded sites are : Da=Dagathie ; Mb=Mbakhana, Th=Thilene, Nd=Ndombo; recently invaded localities are Cr=Croisement Boube, Do=Dodel, Ae=Aere Lao, Lo=Lougue. For the black rat, anciently invaded localities are : Diak=Diakene Wolof, Diat=Diattacounda, Mar=Marsassoum, Tob=Tobor ; recently invaded sites are : Bad=Badi Nieriko, Bou=Boutougoufara, Ked=Kedougou, Sou=Soutouta.

Figure S4. TreeMap view of REVIGO Biological process analyses for the house mouse differentially expressed (DE) genes. Each rectangle represents a single cluster, that are grouped into 'superclusters' of related terms, represented with different colors. The size of the rectangles reflects the frequency of the Gene Ontology (GO) term in this set of DE genes.

Figure S5. Boxplot representing the level of differential expression (in log fold change (log FC)) for immune related and non immune related DE genes identified along the mouse invasion road.

Figure S6. Heatmap of the differentially expressed (DE) genes between the anciently and recently invaded sites of house mouse (*M. musculus domesticus*). The normalized read counts for the expressed genes have been log transformed (log2(values+1)). Heatmap was built in R using heatmap.2 for a) all 364 DE genes and b) 73 immune related genes belonging to over-represented biological processes. The genes (rows) and sites (columns) were clustered using dendrograms built with Ward distance and hierarchical clustering.

Table S1. a) Details of the 18 genes found to be differentially expressed between the anciently and recently invaded sites of *M. musculus domesticus* invasion route using the 4vs4 approach. Genes indicated in bold were found to be differentially expressed with both 8vs8 and 4x4 approaches. b) Details of the 364 genes found to be differentially expressed between the anciently and recently invaded sites of *M. musculus domesticus* invasion route using the 8vs8 approach and found in 85% of the comparisons made using the 4x4 approach. The 73 genes indicated in red are related with immunity and have over-represented GO annotations. The 29 genes underlined and in italics correspond to the immune-related genes with over represented Kegg biological pathways. GO ID corresponds to Gene ontology annotations.

Table S2. a) Details of the 54 genes found to be differentially expressed between the anciently and recently invaded sites of *R. rattus* invasion route using the 4vs4 approach. Genes indicated in bold were found to be differentially expressed with both 8vs8 and 4vs4 approaches. b) Details of the 83 genes found to be differentially expressed between the anciently and recently invaded sites of *R. rattus* invasion route using the 8vs8 approach and found in 85% of the comparisons made using the 4vs4 approach. Genes indicated in bold were found to be differentially expressed with both 8vs8 and 4vs4 approaches. Genes that are underlined are also found to be differentially expressed with the limma package (site as

random effects). Genes indicated in red are related with immunity. GO ID corresponds to Gene ontology annotations.

Differential immune gene expression associated with contemporary range expansion in two invasive rodents in Senegal

Nathalie Charbonnel[1*], Maxime Galan[1], Caroline Tatard[1], Anne Loiseau[1], Christophe Diagne[2,3], Ambroise Dalecky[4], Hugues Parrinello[5], Stephanie Rialle[5], Dany Severac[5] and Carine Brouat[2]

SREP-19-19999A-revised.

Supplementary Figures

Figure S1. Information detailing the quality and quantity of RNA for each pool a) for *Mus musculus domesticus* samples and b) for *Rattus rattus* samples. Bioanalyzer (house mouse) and Fragment analyzer (black rat) profiles are also included in this file.

a) House mouse

Site	Pool ID	Number of individuals	Integrity (gel)	NanoDrop (ng/uL)	260/280	260/230	Volume (uL)	Quantity (ug)	Concentration (ng/uL)	RIN	Quantity (ug)
Dagathie	CB1Da	10	OK	206	2.09	1.70	30	6.2	205	6.9	6.15
Dagathie	CB2Da	8	OK	180	2.10	1.64	30	5.4	166	7.8	4.98
Mbakhana	CB3Mb	10	OK	168	2.09	1.74	30	5.0	155	7.7	4.65
Mbakhana	CB4Mb	10	OK	154	2.06	1.77	30	4.6	155	7.8	4.65
Thilene	CB5Th	8	OK	151	2.07	1.80	30	4.5	152	7.7	4.56
Thilene	CB6Th	8	OK	149	2.08	1.69	30	4.5	168	5.8	5.04
Ndombo	CB7Nd	10	OK	175	2.07	1.68	30	5.3	215	8.1	6.45
Ndombo	CB8Nd	10	OK	131	2.06	1.67	30	3.9	177	8.1	5.31
Croisement Boube	CB9Cr	10	OK	130	2.07	1.56	30	3.9	150	7.0	4.5
Croisement Boube	CB10Cr	10	OK	169	2.09	1.44	30	5.1	201	6.7	6.03
Dodel	CB11Do	10	OK	115	2.09	1.51	30	3.4	139	7.3	4.17
Dodel	CB12Do	8	OK	118	2.07	1.47	30	3.5	145	7.6	4.35
Aere Lao	CB13Ae	10	OK	124	2.06	1.51	30	3.7	106	8.5	3.18
Aere Lao	CB14Ae	10	OK	112	2.09	1.51	30	3.4	105	8.8	3.15
Lougué	CB15Lo	10	OK	129	2.07	1.53	30	3.9	123	8.3	3.69
Lougué	CB16Lo	8	OK	132	2.05	1.49	30	4.0	139	5.6	4.17

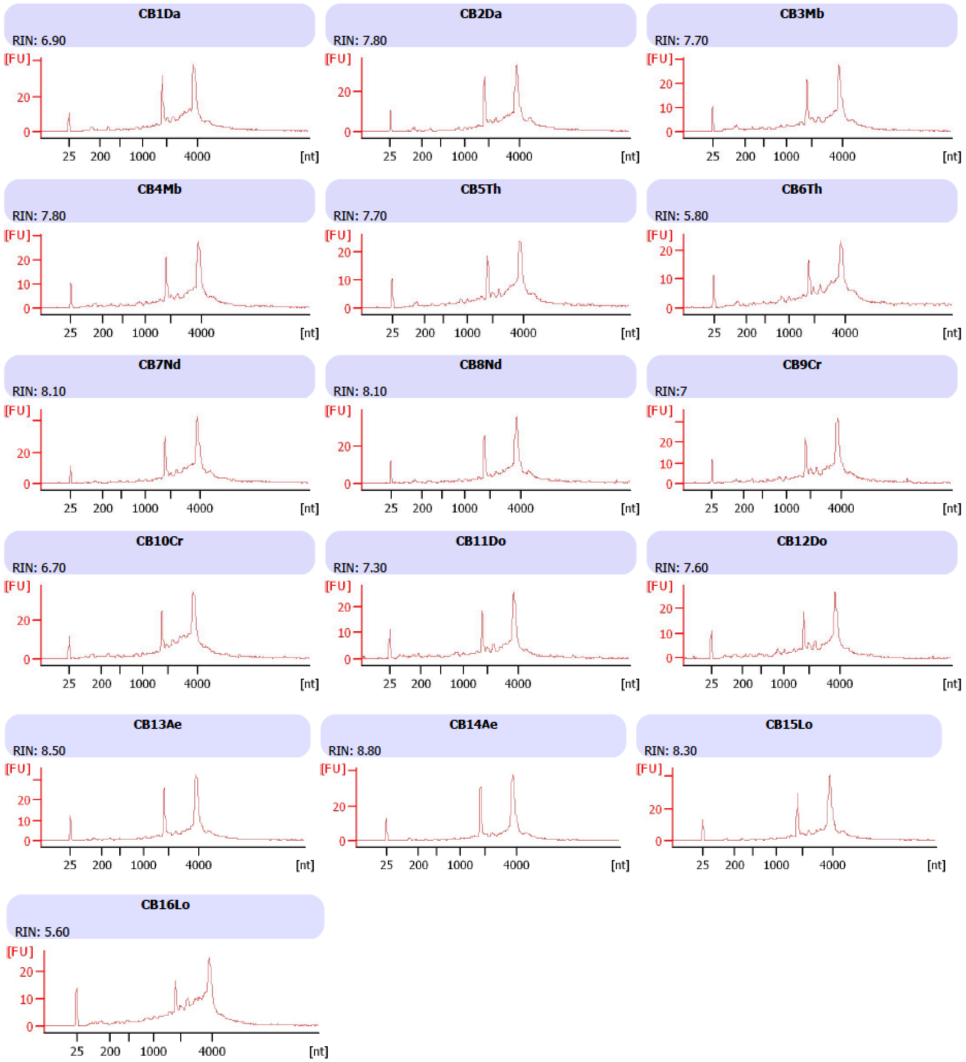
CB1Da
RIN: 6.90
CB2Da
RIN: 7.80
CB3Mb
RIN: 7.70
CB4Mb
RIN: 7.80
CB5Th
RIN: 7.70
CB6Th
RIN: 5.80
CB7Nd
RIN: 8.10
CB8Nd
RIN: 8.10
CB9Cr
RIN:7
CB10Cr
RIN: 6.70
CB11Do
RIN: 7.30
CB12Do
RIN: 7.60
CB13Ae
RIN: 8.50
CB14Ae
RIN: 8.80
CB15Lo
RIN: 8.30
CB16Lo
RIN: 5.60
[FU]
0
10
20
25
200
1000
4000
[nt]

b) Black rat

Site	Pool ID	Number of individuals	Integrity (gel)	NanoDrop (ng/uL)	260/280	260/230	Volume (uL)	Quantity (ug)	Concentration (ng/uL)	RQN	Quantity (ug)
BadiNieriko	RATCB1bad	8	OK	100	2.07	0.52	45	4.5	95.1	8.6	4.28
BadiNieriko	RATCB2bad	8	OK	85	2.07	1.15	45	3.8	81.2	9.7	3.65
Boutougoufara	RATCB3bou	8	OK	112	2.06	0.79	45	5.0	109.6	8.8	4.93
Boutougoufara	RATCB4bou	8	OK	196	2.1	1.1	45	8.8	171.9	9.7	7.74
Kedougou	RATCB5ked	8	OK	215	2.06	1.34	45	9.7	244.3	10	10.99
Kedougou	RATCB6ked	8	OK	144	2.09	0.57	45	6.5	150.5	9.8	6.77
Soutouta	RATCB7sou	10	OK	197	2.07	2	45	8.8	185.3	8.4	8.34
Soutouta	RATCB8sou	10	OK	214	2.08	2.12	45	9.6	221.9	9.0	9.99
DiakeneWolof	RATCB9diak	6	OK	75	2.09	0.15	45	3.4	74.8	10	3.37
DiakeneWolof	RATCB10diak	8	OK	89	2.05	0.78	45	4.0	107.1	9.3	4.82
Diattacounda	RATCB11diat	10	OK	227	2.06	1.74	45	10.2	254.5	9.8	11.45
Diattacounda	RATCB12diat	10	OK	85	2.09	0.27	45	3.8	103.7	/	4.67
Marsassoum	RATCB13mar	10	OK	193	2.05	1.64	45	8.7	215.3	/	9.69
Marsassoum	RATCB14mar	10	OK	156	2.07	1.95	45	7.0	168.6	/	7.59
Tobor	RATCB15tob	6	OK	50	2.06	1.26	45	2.2	59.1	/	2.66
Tobor	RATCB16tob	8	OK	180	2.1	1.82	45	8.1	211.2	/	9.50

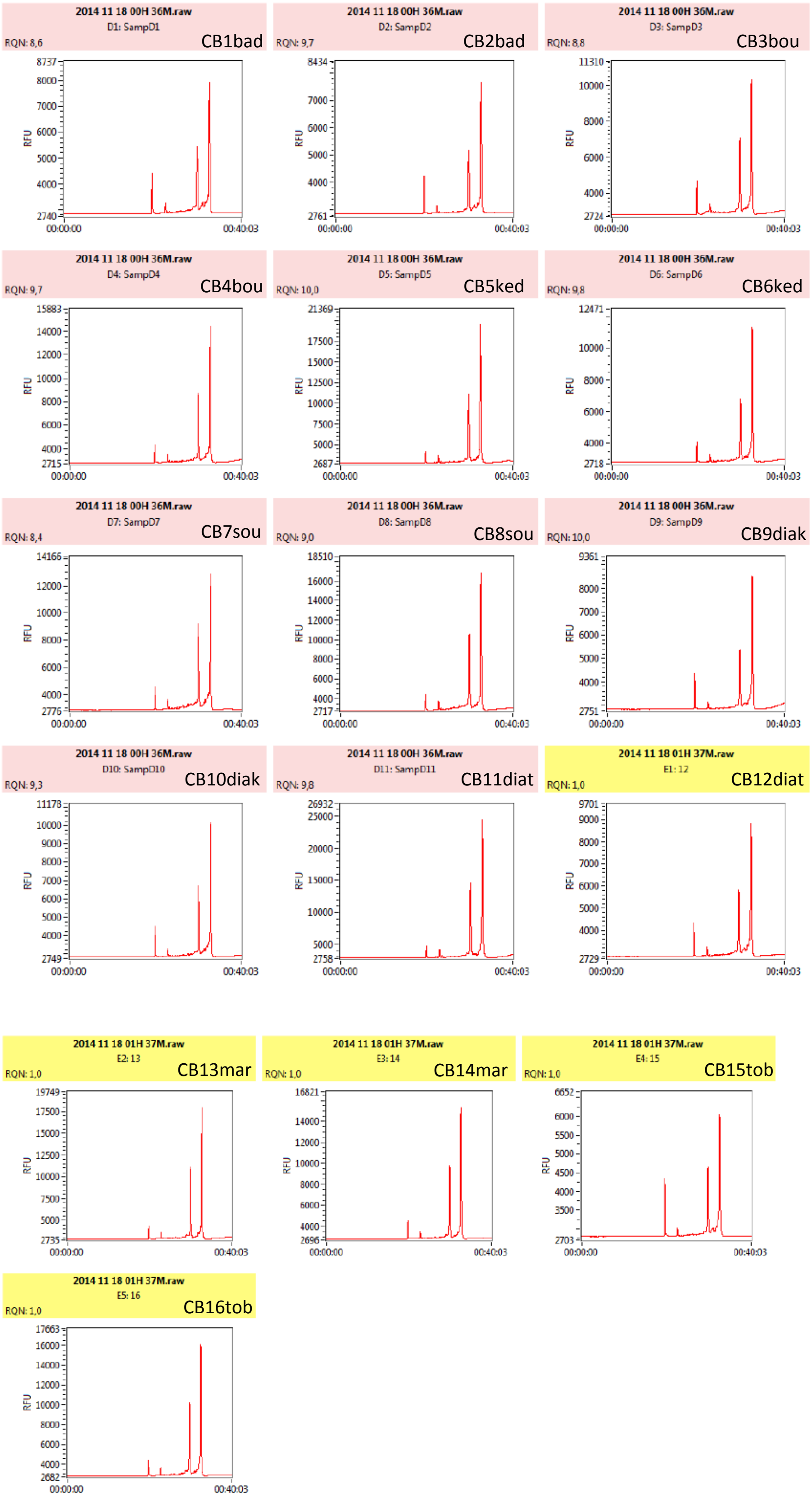
2014 11 18 00H 36M.raw
D1: SampD1
RQN: 8,6
CB1bad
2014 11 18 00H 36M.raw
D2: SampD2
RQN: 9,7
CB2bad
2014 11 18 00H 36M.raw
D3: SampD3
RQN: 8,8
CB3bou
2014 11 18 00H 36M.raw
D4: SampD4
RQN: 9,7
CB4bou
2014 11 18 00H 36M.raw
D5: SampD5
RQN: 10,0
CB5ked
2014 11 18 00H 36M.raw
D6: SampD6
RQN: 9,8
CB6ked
2014 11 18 00H 36M.raw
D7: SampD7
RQN: 8,4
CB7sou
2014 11 18 00H 36M.raw
D8: SampD8
RQN: 9,0
CB8sou
2014 11 18 00H 36M.raw
D9: SampD9
RQN: 10,0
CB9diak
2014 11 18 00H 36M.raw
D10: SampD10
RQN: 9,3
CB10diak
2014 11 18 00H 36M.raw
D11: SampD11
RQN: 9,8
CB11diat
2014 11 18 01H 37M.raw
E1: 12
RQN: 1,0
CB12diat
2014 11 18 01H 37M.raw
E2: 13
RQN: 1,0
CB13mar
2014 11 18 01H 37M.raw
E3: 14
RQN: 1,0
CB14mar
2014 11 18 01H 37M.raw
E4: 15
RQN: 1,0
CB15tob
2014 11 18 01H 37M.raw
E5: 16
RQN: 1,0
CB16tob
RFU
00:00:00
00:40:03

Figure S2. Schematic representation of the two approaches defined to analyze gene differential expression between anciently and recently invaded sites.

Sites are described with two pools of individuals, and these two pools are considered as biological replicates of a given site (solid circle and dashed circle). Red= Anciently invaded; Green= Recently invaded. L = site (geographic locality).

I- The 4vs4 approach
It is based on the comparison of four anciently invaded sites (a single biological replicate is picked up randomly for each site) versus four recently invaded sites (a single biological replicate is picked up randomly for each site).
There are 256 possibilities (128 and their complementary)

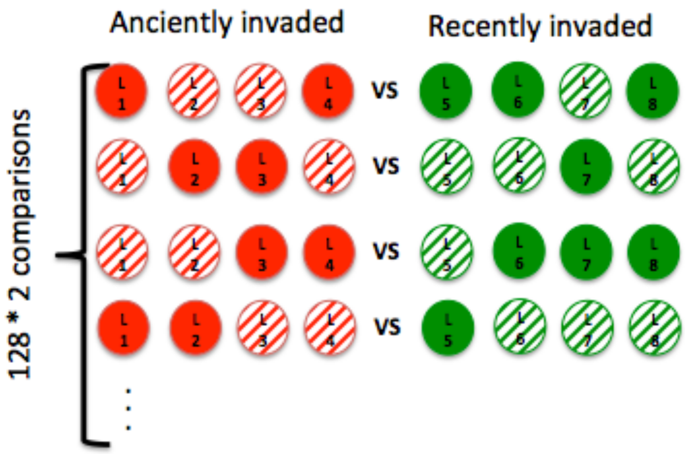

For example here, the first possibility (Line 1) is
Site1 Pool1, Site2 Pool2, Site3 Pool2, Site4 Pool 1 (anciently invaded) Versus Site5 Pool1, Site6 Pool 1, Site7 Pool2, Site8 Pool1 (recently invaded)

The complementary possibility (Line 2) is
Site1 Pool2, Site2 Pool1, Site3 Pool1, Site4 Pool 2 (anciently invaded) Versus Site5 Pool2, Site6 Pool2, Site7 Pool1, Site8 Pool2 (recently invaded)

Another possibility (Line 3) is
Site1 Pool2, Site2 Pool2, Site3 Pool1, Site4 Pool 1 (anciently invaded) Versus Site5 Pool2, Site6 Pool 1, Site7 Pool1, Site8 Pool1 (recently invaded)

The complementary possibility (Line 4) is
Site1 Pool1, Site2 Pool1, Site3 Pool2, Site4 Pool 2 (anciently invaded) Versus Site5 Pool1, Site6 Pool2, Site7 Pool2, Site8 Pool2 (recently invaded)

Etc…

II- The 8vs8 approach
It is based on the comparison of the eight pools describing the anciently invaded sites vs the eight pools describing the invaded sites.

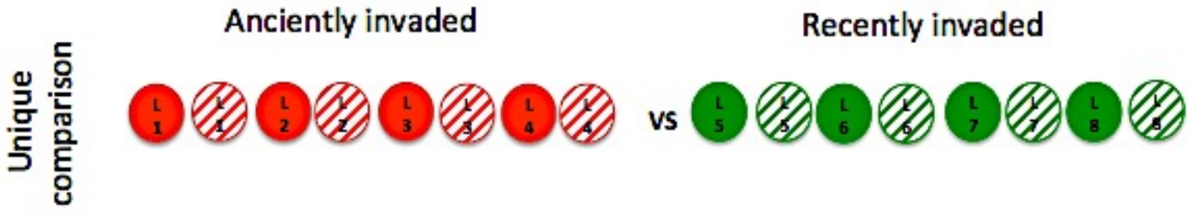

Supplementary Figure S3. Pairwise comparisons of the RLE-normalized total number of reads between libraries for a) the house mouse and b) the black rat. Da=Dagathie ; Mb=Mbakhana, Th=Thilene, Nd=Ndombo, Cr=Croisement Boube, Do=Dodel, Ae=Aere Lao, Lo=Lougue. Bad=Badi Nieriko, Bou=Boutougoufara, Diak=Diakene Wolof, Diat=Diattacounda, Ked=Kedougou, Mar=Marsassoum, Sou=Soutouta, Tob=Tobor.

a)

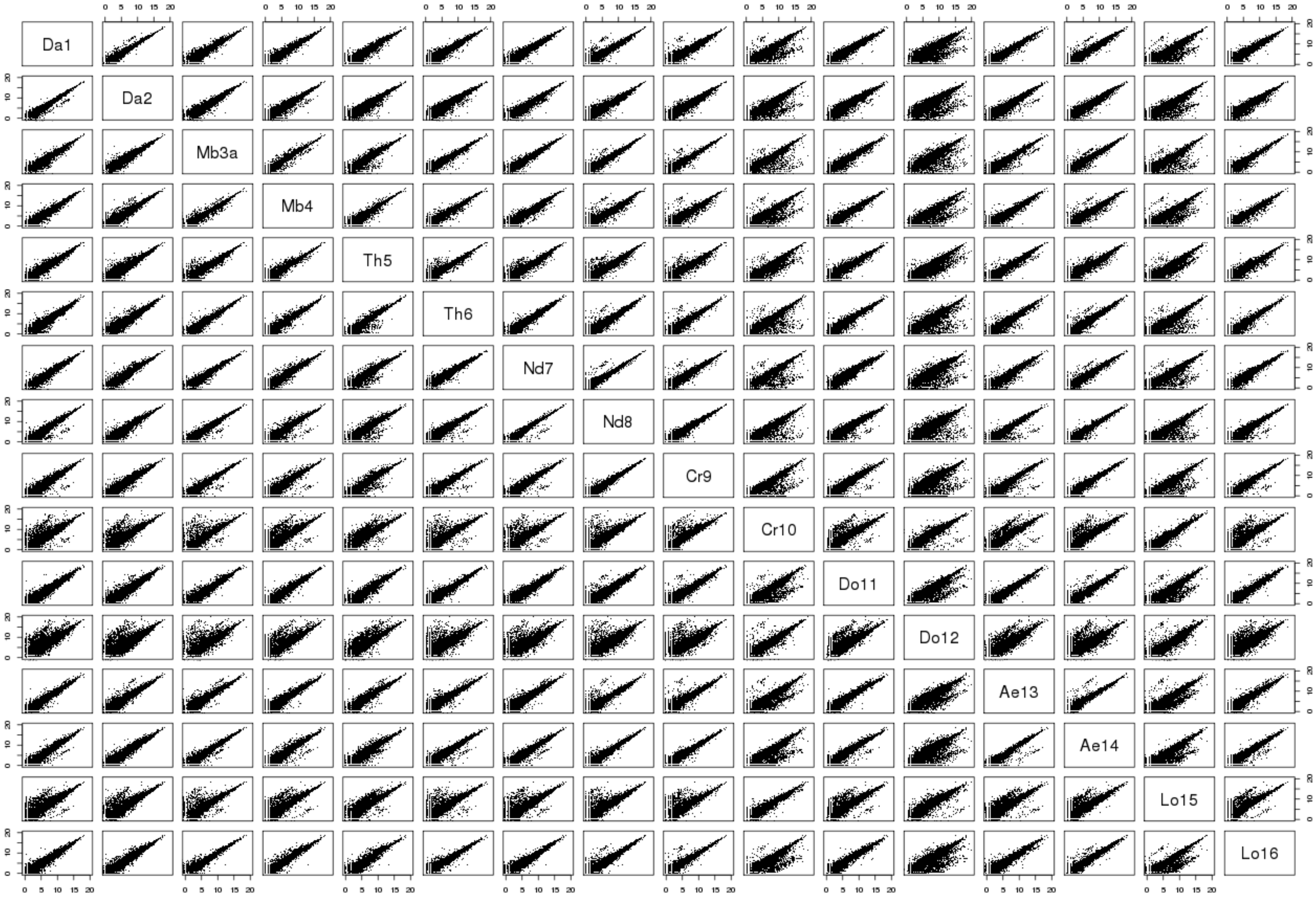
Da1
Da2
Mb3a
Mb4
Th5
Th6
Nd7
Nd8
Cr9
Cr10
Do11
Do12
Ae13
Ae14
Lo15
Lo16

b)

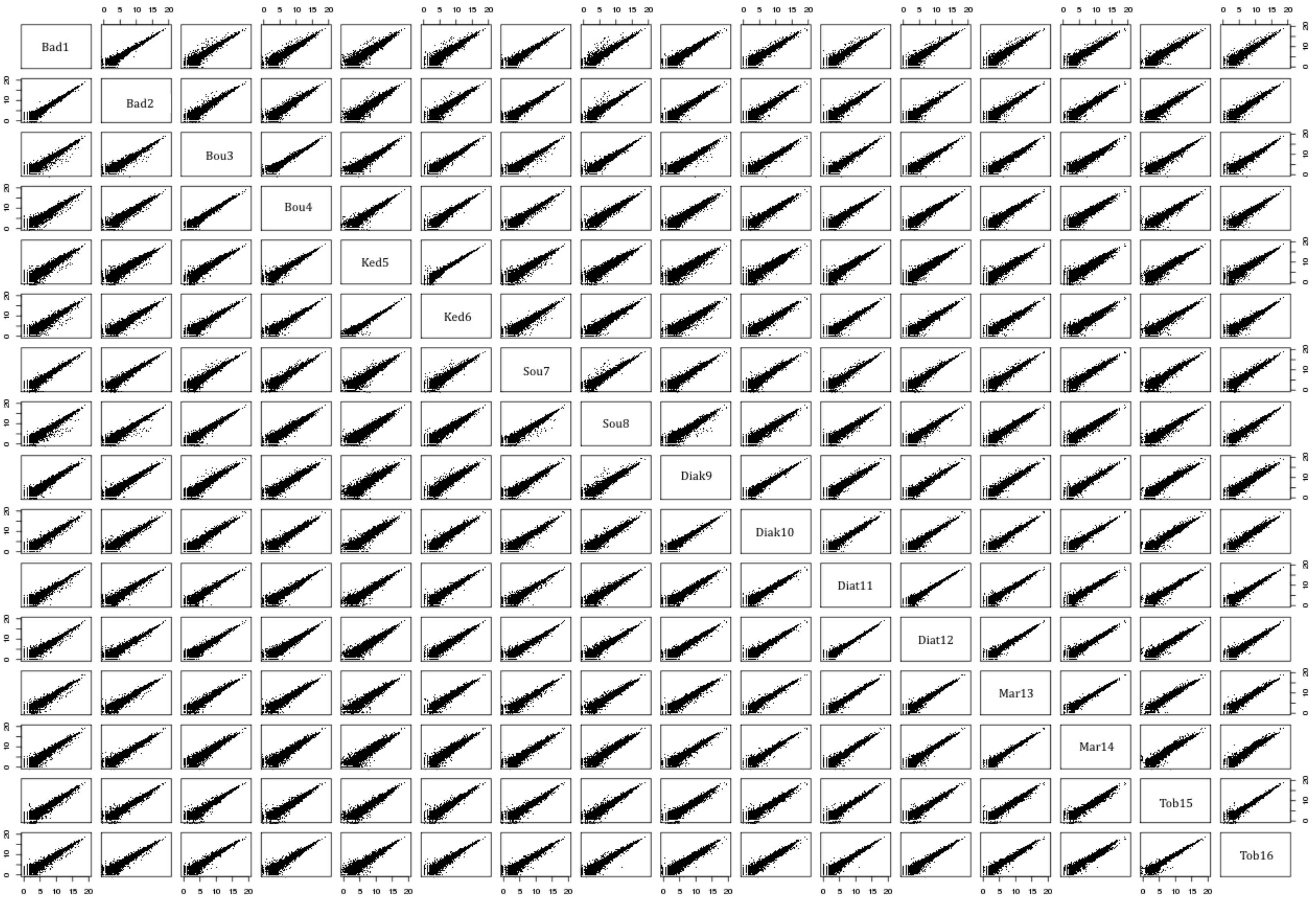

Bad1
Bad2
Bou3
Bou4
Ked5
Ked6
Sou7
Sou8
Diak9
Diak10
Diat11
Diat12
Mar13
Mar14
Tob15
Tob16

Supplementary Figure S4. TreeMap view of REVIGO Biological Process analyses for the house mouse differentially expressed (DE) robust genes. Each rectangle represents a single cluster. Large groups represent 'superclusters' of related gene ontology (GO) terms, and they are represented with different colours. The size of the rectangles reflects the frequency of the GO term in this set of DE genes.

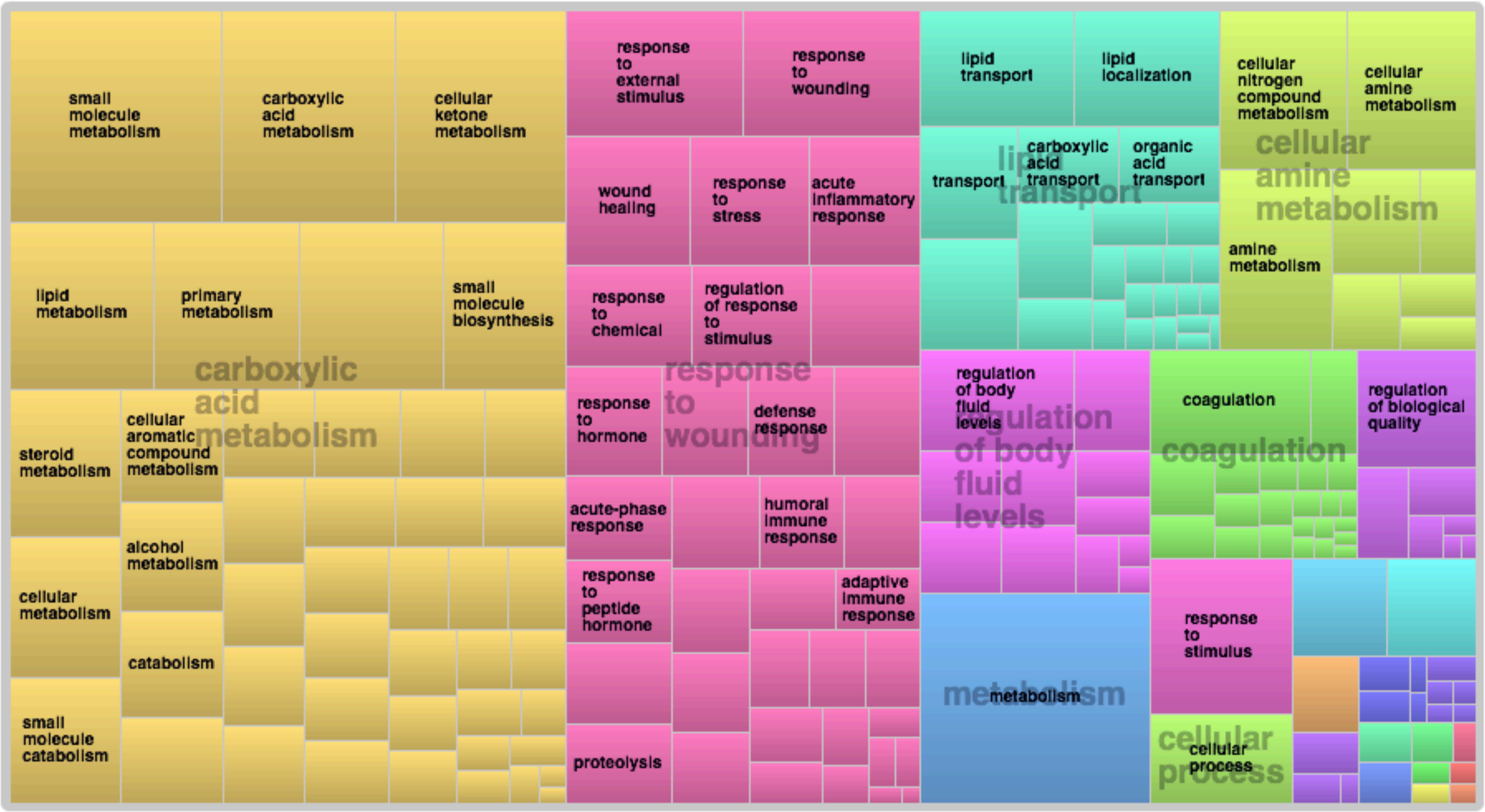

Supplementary Figure S5. Boxplot representing the level of differential expression (in log fold change (log FC)) for immune related and non immune related DE genes identified along the mouse invasion road.

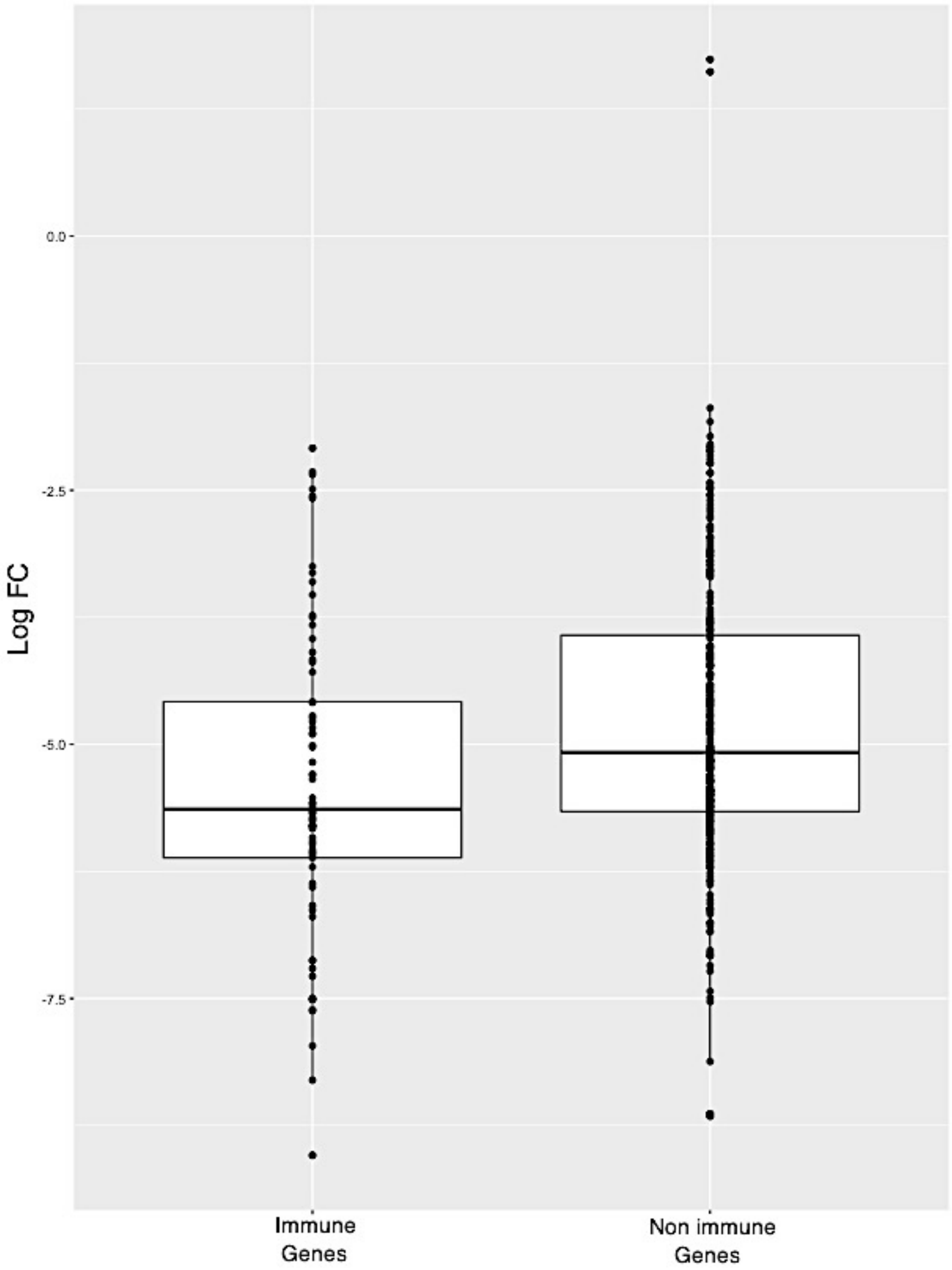

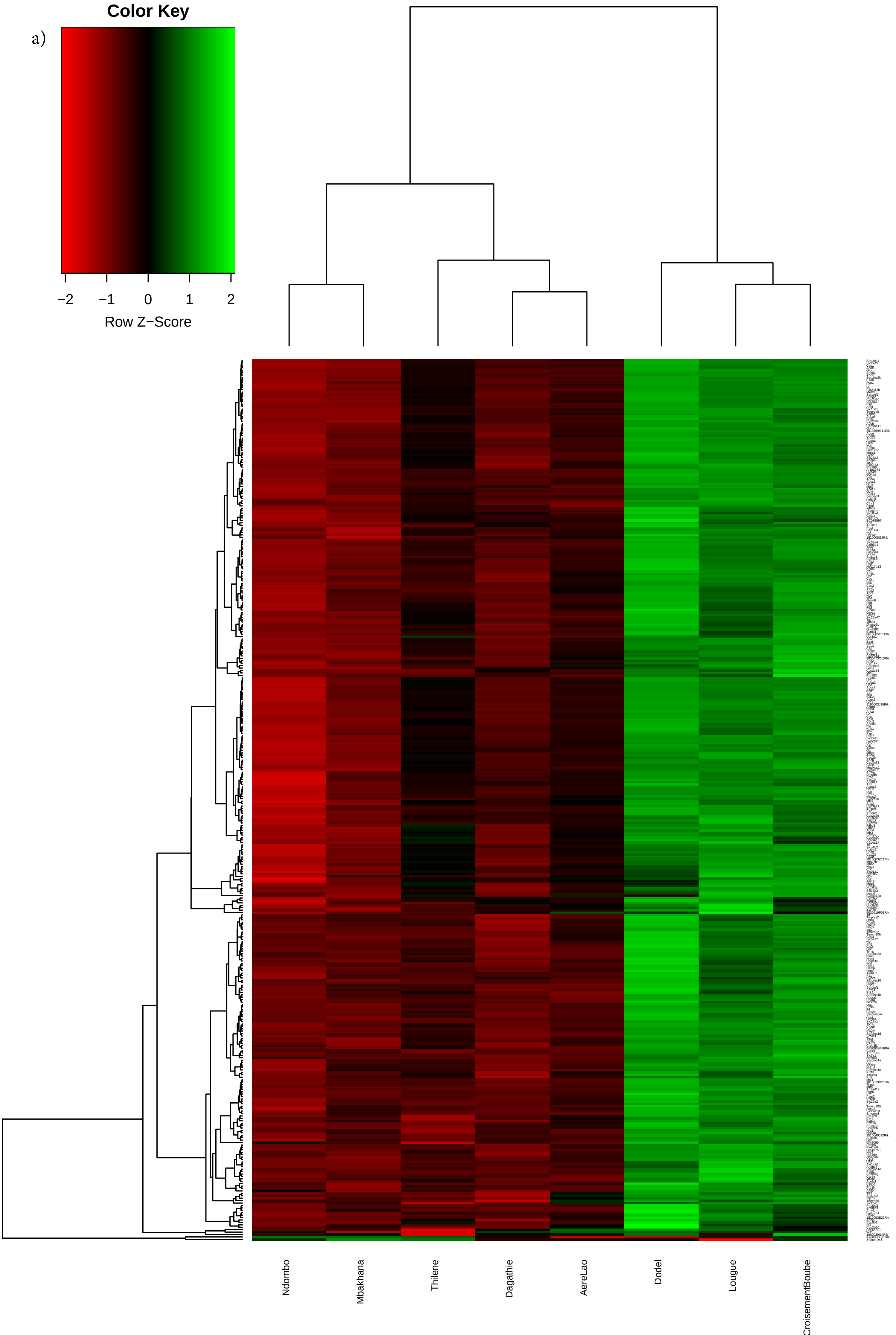
a)
Color Key
−2
−1
0
1
2
Row Z−Score
Ndombo
Mbakhana
Thilene
Dagathie
AereLao
Dodel
Lougue
CroisementBoube

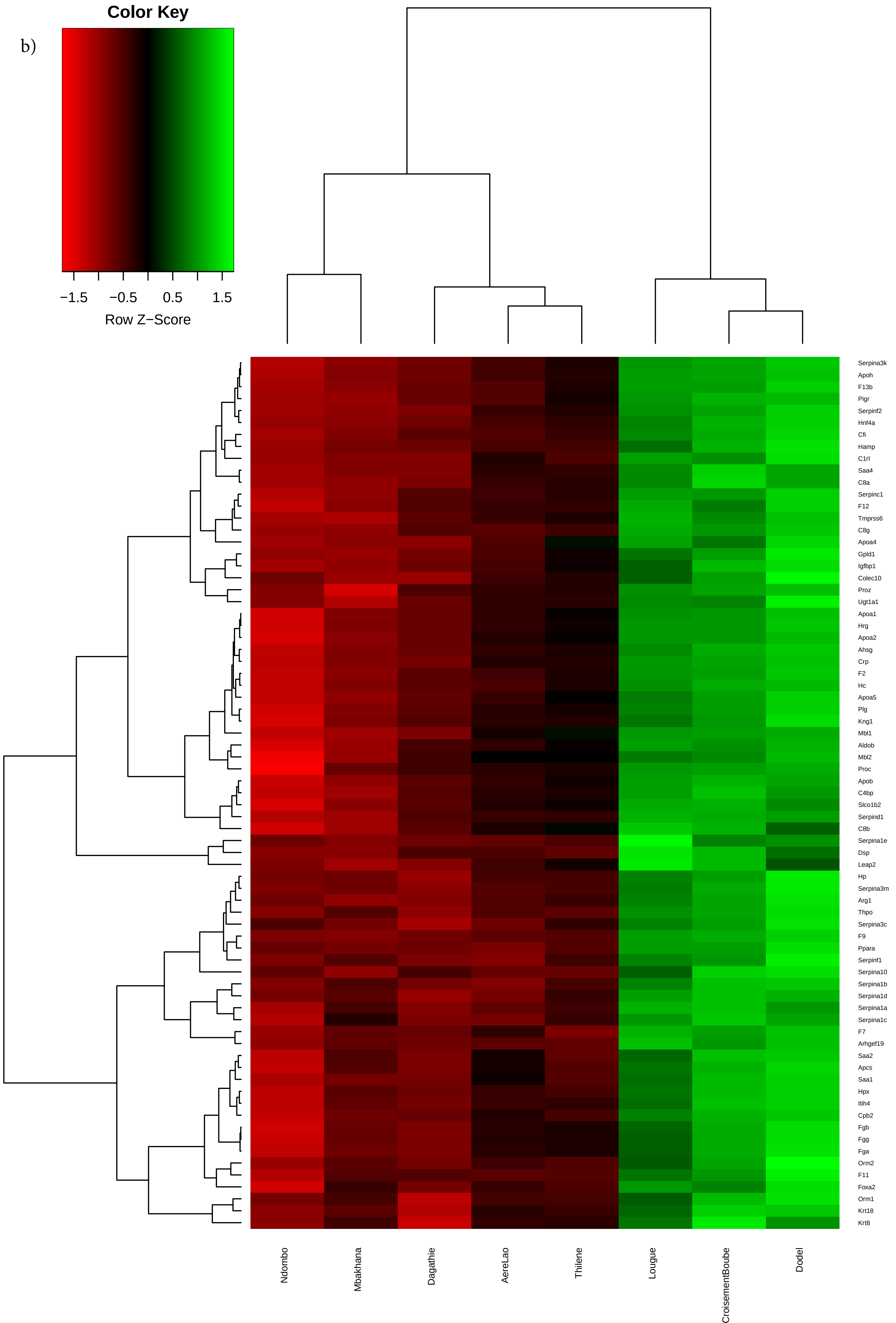

b)
Color Key
−1.5
−0.5
0.5
1.5
Row Z−Score
Serpina3k
Apoh
F13b
Pigr
Serpinf2
Hnf4a
Cfi
Hamp
C1rl
Saa4
C8a
Serpinc1
F12
Tmprss6
C8g
Apoa4
Gpld1
Igfbp1
Colec10
Proz
Ugt1a1
Apoa1
Hrg
Apoa2
Ahsg
Crp
F2
Hc
Apoa5
Plg
Kng1
Mbl1
Aldob
Mbl2
Proc
Apob
C4bp
Slco1b2
Serpind1
C8b
Serpina1e
Dsp
Leap2
Hp
Serpina3m
Arg1
Thpo
Serpina3c
F9
Ppara
Serpinf1
Serpina10
Serpina1b
Serpina1d
Serpina1a
Serpina1c
F7
Arhgef19
Saa2
Apcs
Saa1
Hpx
Itih4
Cpb2
Fgb
Fgg
Fga
Orm2
F11
Foxa2
Orm1
Krt18
Krt8
Ndombo
Mbakhana
Dagathie
AereLao
Thilene
Lougue
CroisementBoube
Dodel
